# Comparison of pathologic characteristics of breast cancer in younger and older women

**DOI:** 10.22088/cjim.10.1.42

**Published:** 2019

**Authors:** Reza Yazdani-Charati, Karimollah Hajian-Tilaki, Majid Sharbatdaran

**Affiliations:** 1 Student Research Committee, Babol University of Medical Sciences, Babol, Iran; 2 Social Determinants of Health Research Center, Health Research Institute, Babol University of Medical Sciences, Babol, Iran; 3 Cancer Research Center, Health Research Institute, Babol University of Medical Sciences, Babol, Iran

**Keywords:** Breast cancer, younger women, tumor characteristics, TNM classification

## Abstract

**Background:**

Breast cancer is the most common invasive cancer with high mortality in women all around the world. The present evidence shows that younger patients have poor survival. Thus, the aim of this study was to compare the pathologic characteristics of breast cancer in women younger than 40 years compared with older.

**Methods:**

This is a cross-sectional study which contains 681 patients with a confirmed diagnosis of breast cancer, who referred to Babolsar Shahid Rajaei Hospital as a referral cancer therapeutic center in the North of Iran. The data included age, residence area, occupation, location, histopathologic characteristics of the tumor, TNM classification and staging.

**Results:**

The mean age (SD) of patients was 49.7 (11.9) years, of which 19.5% were under 40. Ductal carcinoma was the most common histopathologic type (90.0%) but patients at a younger age had a higher incidence of lobular and other rare carcinoma compared to the older ones (P=0.04). The younger had a greater tumor size (P=0.01), lymphatic node involvement (P=0.04) and higher staging (P=0.004). The younger age was not associated with positive estrogen/progesterone receptors.

**Conclusion:**

These findings indicated more aggressive tumor characteristics and serious breast cancer in women less than 40 years compared with older ones.

Breast cancer is a major public health problem and the most common aggressive cancer in women with a high rate of mortality worldwide (1-4). It was estimated that 1.8 million new cases of breast cancer were diagnosed in 2015 all over the world (5). Also, the American Cancer Society reported that 231840 new cases of invasive and 60290 nonaggressive breast tumor were diagnosed in American women in 2015 (6). Annually, roughly 40290 American women die from breast cancer that is attributed to 3% of all deaths and women living in the United States has 12.3% lifetime risk i.e. 1 in 8 women suffers from breast cancer in her life span (6). According to the International Agency of Cancer Research, about 10980 patients of new breast cancer were diagnosed in the Islamic Republic of Iran in 2015 (5). In fact, breast cancer includes 18.9% of all cases of cancer incidence among Iranian women. A perspective view of the demographic characteristic of breast cancer incidence in Iranian women is that the patients’ age at diagnosis is, on average, about 10 years younger than western counterparts (7). The multiple risk factors such as family history, reproductive behaviors, lifestyle-related factors like obesity, smoking, low physical activity and exposure to radiation, stress, and anxiety were reported as determinants of its incidence (7-10).

Its prognosis depends on the type of therapeutic agents received, staging and pathological characteristics of tumor (tumor size, node involvement and presence of metastasis (TNM) and the demographic profiles that may have all predicted the survival changing in lifestyles and reproductive behaviors in recent decades (7, 9, 10 and prognosis (11, 12). Despite the low incidence of breast cancer in younger women, delay in diagnosis is possible; on the other hand, the poor prognosis in younger patients raised the question whether this is designated by the pathological characteristics of tumor or not. In spite of changes in lifestyles and reproductive behaviors in recent decades (7, 9, 10) which has led to an increase in the incidence of breast cancer in Iranian women particularly in the North, the South of Caspian sea, the comparative data of pathologic characteristics of younger and older women is sparse in this region. Thus, the objective of this study was to compare the pathologic findings between women younger than 40 years and older. This new information would help public health managers for prevention, screening, and promotion of survival and also would enhance the knowledge of the general population in promotion of self- care behaviors. 

## Methods

This cross-sectional study was conducted in Babolsar Shahid Rajaie Hospital, as a referral center for cancer radio-chemotherapy in the North of Iran. A total of 681 patients with pathologically confirmed breast cancer were entered in the study that was treated from March 2005 to December 2014. Male breast cancer patients and patients who had no pathologic reports and those which their charts were inaccessible were excluded from the study. Through patients’ names and their charts’ numbers, their hospital charts were extracted. The demographic characteristics, clinical and histopathological variables were also extracted from patients’ charts and the study checklists were fulfilled. The data included age, residence area, the presence of estrogen and progesterone receptors, type of tumors histopathology, breast location, tumor size, nodal involvement, the presence of metastasis at diagnosis were collected. In all records, T, N, M stages were determined according to TNM classification. Estrogen and progesterone receptors (ER and PR) expression were assessed by enzyme immuno-histopatho chemistry immunoassay as positive or negative. The stage was calculated based on the clinical definition of TNM classification. The protocol of this study was approved by the Ethics Committee of Babol University of Medical Sciences (MUBABOL.REC.1394.225).

**Statistical Analysis: **We used SPSS software Version 18.0 for data analysis. The patients were categorized into two groups (less than 40 and ≥40 years). The descriptive statistics were presented as mean±SD for quantitative data and the frequency and percentage by the cross table for categorical data. The distribution of the size of the tumor, nodal involvement and the presence of metastasis and staging and histopathology type were compared between two groups of ages. The Chi-square test was performed to determine the association between pathologic findings and age group and the p-values less than 0.05 were considered as significant level. In addition, the logistic regression model was applied to estimate the odds ratio and its 95% confidence interval of younger age (<40 years) versus older in association with tumor characteristics as outcome variables. In the logistic regression model, the presence of tumor size of T2 or greater, the presence of nodal involvement, the presence of metastasis, the stage of 2 or higher, PR positive and ER positive were considered as outcomes of interest.

## Results

The mean age (±SD) of patients was 49.7±11.9 years (ranged from 17 to 95 years). The majority of patients (80.5%) was ≥40 years versus 19.5% at age <40 years. The most common prevalent age group was 40-49 years (33.0%). Most patients were residents of the urban area (65.6%) and housewives (87.3%) and only 12.6% were either working or retired. 

About 46.7% of cases, the breast malignancy was located on the right side of the breast and 51.2% on the left side and in only 2.0% of cases, malignancy was bilateral. Table 1 shows that among 526 women with known status of hormone receptors, older women (≥40 years) compared with younger (<40 years) had a greater proportion of positive progesterone receptor (74.1% versus 69.6%), but the difference did not appear to be significant (P=0.63). A similar proportion of women had positive estrogen receptor in older age compared with younger (74.1% versus 68.6%, P=0.26). Table 2 indicates the distribution of TNM classification and histological type according to age group. Ductal carcinoma was the most common prevalent histological type (90.0%) while the relative frequency of lobular carcinoma was about 5.9% and the other rare breast malignancies were 4.1%. Lobular carcinoma and other rare histological types such as medullary, lymphoma, angiosarcoma but not ductal carcinoma, were more significantly common in younger age compared with older women (P=0.04). In both age groups, tumor size of T2 (55%) was the most frequent and overall, younger women had a greater tumor size than older (P=0.01). Moreover, younger women had a significantly greater proportion of nodal involvement (P=0.04) but the association between age and the presence of metastasis did not appear to be significant (P=0.50). 

**Table 1:** The positive and negative progesterone (PR) and estrogen (ER) receptors in breast cancer patients with age groups

**Hormone receptors**	**All** * **N (%)**	**<40 y** **N (%)**	**≥40 y** **N (%)**	**P-value**
PR+ PR-	385(73.2) 141(26.8)	71(69.6 31(35.4)	314(74.1) 110(25.9)	0.63
ER+ ER-	384(73.0) 142(27.0)	70 (68.6) 32(31.4)	314(74.1) 110(25.9)	0.26
All	526 (100)	102(100)	424 (100)	

* In 155 patients’ charts, the data of hormone receptors were unknown.

**Table 2:** The tumor characteristics (TNM classification) and histopathology of breast cancer patients with respect to age groups

**TNM Classification&** **Histopathology**		**All** **N (%)**	**<40 y** **N (%)**	**≥40 y** **N (%)**	**P-value**
Tumor size	T1 T2 T3 T4	149(23.3) 352(55.1) 113(17.7) 25 (3.9)	17(13.9) 72(59.0) 30(24.6) 3 (2.5)	132(25.5) 280(54.2) 83 (16.1) 22 (4.3)	0.01
Node Involvement	N0 N1 N2 N3	271(42.5) 256(40.2) 85 (13.3) 25 (3.9)	42(34.3) 57(46.4) 21(17.2) 2 (1.6)	226(42.5) 199(38.6) 64 (12.4) 23 (4.5)	0.04
Metastasis	M0 M1	588(91.3) 56 (8.7)	116(92.8) 9 (7.2)	472(90.9) 47 (9.1)	0.50
Histopathology	Ductal Lobular Others*	591 (90) 39 (5.9) 27 (4.1)	108(84.4) 8 (6.3) 12 (9.4)	483(91.3) 31 (5.9) 15 (2.8)	0.04

* Medullary, lymphoma, angiosarcoma

Regarding TNM classification, on the 633 women with known status of all TNM characteristics, the clinical staging was determined and table 3 shows 46.4% of patients (21.7% versus 28.2% in older and younger respectively) were diagnosed at stage III (IIIA, IIIB, and IIIC) overall. Only 5.8% of younger and 18.2% of older women were diagnosed at stage I. The more advanced stage was observed in younger women and this was statistically significant (P=0.004). Moreover, in our findings, lobular carcinoma and other rare carcinoma had a greater chance of being metastatic (P=0.04) but the association of histological type with stage and the presence of estrogen receptor was not observed with statistical significance (P=0.10, P=0.07, respectively). Table 4 presents that odds ratio and its 95% confidence interval of younger women versus older in association with tumor characteristics. A significant association was found in relation with the stage of ≥ 2 (OR=3.63, 95%CI: 1.64-8.02), tumor size of≥T2 (2.13, 95%CI: 1.23-3.68) and nodal involvement (OR=1.55, 95%CI: 1.03-2.35), while other characteristics did not appear to be significant.

**Table 3:** The staging status of breast cancer patients according to age groups

**Stage**	**All** * **N (%)**	**<40 y** **N (%)**	**≥40 y** **N (%)**	**P-value**
I	100 (15.8)	6 (5.8)	93 (18.2)	0.004
IIA	56 (8.8)	35 (28.9)	127 (24.8)	
IIB	162 (25.6)	36 (29.8)	134 (26.2)	
IIIA	10 (26.9)	29 (24.0)	72 (14.1)	
IIIB	101 (16.0)	3 (2.5)	19 (3.7)	
IIIC	22 (3.5)	2 (1.7)	20 (3.9)	
IV	22 (3.5)	9 (7.4)	47 (9.2)	
All	633 (100)	121 (100)	512 (100)	

* In 48 cases, the stage was not determined because of missing data of TNM status.

**Table 4:** The odds ratio (OR) and its 95% confidence interval (CI) of younger age versus older in association with tumor characteristics as outcome variables

**Tumor characteristics **	**OR** **(95% CI)**	**P-value**
Tumor size ≥T2 vs. T1	2.13 (1.23, 3.68)	0.007
Nodal involvement vs. not	1.55 (1.03, 2.35)	0.035
Stage≥2 vs. Stage 1	3.62 (1.64, 8.02)	0.002
Presence of metastasis vs. not	0.80 (0.38, 1.67)	0.50
PR^+^ vs. PR^-^	0.80 (0.50, 1.29)	0.36
ER^+ ^vs. ER^-^	0.76 (0.47, 1.21)	0.24

## Discussion

Our findings show that the mean age of breast cancer incidence was 49.7 years and 19.5% were diagnosed at age less than 40 and the most prevalent age group was 40-49 years. This issue is in contrast to the patients in the United States with developed breast cancer, which their mean age was 61 years (6). In the study by Alieldin et al (14) and also Abdullateef et al. (15) reported among Egyptian and Iraqi women, respectively, the most common age group was 40-49 years of which is rather similar to our findings, but in the study by Colleoni et al., only 2% of patients were at age <40 years in European women (16). Additionally, women <=40 years comprise roughly 5% of all new cases in the United States (17). While our findings and other reports from Iranian female breast cancer (7, 18) indicate that the experience of Iranian women was about one decade lower than western counterparts at the time of diagnosis.

In the present study, ductal carcinoma was the most prevalent histological type. This is in accordance with other reports in different regions of Iran (7). Abdollateef et al also found a higher proportion of ductal carcinoma (98%) than ours and a very low prevalence of lobular carcinoma (15). While in our findings, the proportion of lobular carcinoma and other rare breast malignancy were more common in younger age. In contrast, others reported a greater proportion of lobular carcinoma in older age (18, 19). The differences were mainly explained by ethnic variation and the differences in socioeconomic characteristics and nutritional behaviors.

In our study, the negative estrogen receptor was tended to be greater in younger women but the difference was not statistically significant. This might be due to the lack of sufficient sample size because of some missing data on hormone receptor status in our study samples that preclude achieving a reasonable statistical power for detection of its association. However, some reports showed that the presence of negative estrogen receptor in younger women was significantly greater than the older ones (19, 20). Considering the fact that the expression of hormone receptors would have a favorable response to hormonal therapy (6), therefore, this would be an evidence for poor prognosis of disease in younger patients. Thus, the younger patients with a high rate of negative estrogen and progesterone receptors, need aggressive therapeutic agents such as chemotherapy instead of hormonal therapy alone (20). In our findings, according to tumor size, overall, the highest proportion of patients were at T2 (2-5 cm) in TNM classification, while the younger patients had a greater experience with a higher tumor size. This result was also inconsistent with other reports (18, 21). It seems the probability of untouchable tumor with the size of 3-4 cm increases in younger ones. This untouchable size by patients or nurses would help subsequent follow up for further workup for diagnosis. But the higher prevalence of greater tumor size in younger women is as an indicator of increased risk of malignancy (19). Additionally, in our result, the proportion of nodal involvement was greater significantly in younger. This finding also is in accordance with other reports (19, 20). This similarity of results highlights an evidence for poor prognosis of breast cancer for younger women.

 In the present study, the younger women had significantly higher stage at diagnosis than older ones and a few younger patients were diagnosed in stage I compared to the older ones. In both groups of age, the majority of patients were diagnosed at stage II and III. Besides, this result is rather similar to those reported in other studies (15, 20, 22). Nonetheless, in our finding, a smaller proportion of patients were diagnosed at stage I than other studies. This is perhaps because of lack of mass screening program among Iranian women (23). This study may have some limitations for generalizability of results. Because it is based on existing data in hospital charts. Some hospital charts have no data on pathologic findings and hormone receptor status and a few charts were inaccessible. This may preclude generalizability of findings. Nevertheless, the patients without pathologic reports that were excluded from the study and those who had this report to meet the inclusion criteria for recruitment might not differ in terms of pathologic characteristics and severity of diseases. This may reasonably assume to occur non-differential pattern since there was no systematic intention for chart selection. In addition, the relatively large sample size included in the study minimizes the sampling variations and random errors on the estimate of proportion indexes to achieve a desired power of statistical test in the comparison between two groups under study.

In conclusion the results indicate that breast cancer has a high incidence among Iranian women less than 40 years old and thus, the mean age of its incidence is a decade lower compared with western counterparts. The younger patients have a higher tumor size and greater nodal involvement than older 40 years and thus their diagnosis occurs at a higher stage in younger age. Therefore, younger women were involved with more aggressive tumor and thus poor prognosis.
